# Dispersive Solid–Liquid Extraction Coupled with LC-MS/MS for the Determination of Sulfonylurea Herbicides in Strawberries

**DOI:** 10.3390/foods8070273

**Published:** 2019-07-22

**Authors:** Nho-Eul Song, Dong-Ho Seo, Ji Yeon Choi, Miyoung Yoo, Minseon Koo, Tae Gyu Nam

**Affiliations:** Food Analysis Center, Korea Food Research Institute, Wanju 55365, Korea

**Keywords:** dispersive solid-liquid extraction, sulfonylurea herbicides, QuEChERS, strawberry, cleanup

## Abstract

The monitoring of food quality and safety requires a suitable analytical method with simultaneous detection in order to control pesticide and herbicide residues. In this study, a novel analytical method, referred to as “dispersive solid–liquid extraction”, was applied to monitor seven sulfonylurea herbicides in strawberries. This method was optimized in terms of the amount of C_18_ and the volume of added water, and it was validated through satisfactory linearities (*R*^2^ > 0.99), recoveries of 70% to 84% with acceptable precisions, and limits of quantification lower than the maximum residue limits for the seven sulfonylurea herbicides in strawberries. The cleanup efficiency of the dispersive solid–liquid extraction technique was compared to that of the QuEChERS- (“quick, easy, cheap, effective, rugged and safe”) based method with dispersive solid phase extraction. The recoveries of the former were found to be comparable to those involving QuEChERS C_18_ cleanup (recoveries of 74%–87%). The method was used to determine sulfonylurea herbicide residues in ten strawberry samples. None of the samples had herbicide residues higher than that of limit of quantifications (LOQs) or maximum residue limits (MRLs). The results suggest that the dispersive solid–liquid extraction method combined with liquid chromatography-tandem mass spectrometry (LC-MS/MS) is effective for the analysis of sulfonylurea herbicide residues in strawberries.

## 1. Introduction

With ever-tightening regulations that govern the maximum residue limits (MRLs) of pesticides and herbicides in foods, issues associated with their residues and food safety have received significant attention. Multi-residue analysis techniques are widely used to monitor food quality and safety [1]. While the multi-residue analysis of pesticides is capable of determining trace components in food matrices, it requires effective sample preparation, including extraction and cleanup steps, in order to eliminate interference (pigments, lipids, etc.) present in real samples [2,3]. The fact that pesticide multi-residues are associated with a broad spectrum of chemical and physical properties, provides challenges that require suitable extraction and cleanup procedures in order to detect these analytes with satisfactory accuracies.

Sulfonylurea herbicides are used to control broadleaf weeds and annual grasses in agricultural crops. The European Union (EU) [4] and Korea [5] have set MRLs for most sulfonylurea herbicide residues in strawberries at 0.01 mg/kg; however MRLs have not been set for all sulfonylurea herbicides. Therefore, controlling these herbicide residues in foods through monitoring is important for consumer safety. The QuEChERS (“quick, easy, cheap, effective, rugged, and safe”) sample preparation technique coupled with MS and MS/MS is a widely accepted methodology for most pesticides, with the exception of nonpolar pesticides in food materials [6]. Unfortunately, the original QuEChERS method provided relatively weak recovery values (<70%) for sulfonylurea herbicides [7,8]. To overcome this issue, several sample preparation techniques, including solid phase extraction (SPE) using Chem Elut SPE cartridges [9] or a mini-column packed with oxidized carbon nanotubes [10], magnetic-SPE using multiwalled carbon nanotubes [11], dispersive SPE (d-SPE) using C_18_ and graphitized carbon black (GCB) [12], dispersive liquid–liquid microextraction (DLLME) [13], matrix solid phase dispersion followed by DLLME [14], and stir bar sorption extraction [15], have been studied and validated. Lee et al. [8] reported that a modified QuEChERS method involving C_18_ cleanup after extraction with a citrate buffer provided the best recoveries for some sulfonylurea herbicides in brown rice and rice straw. Kaczyński and Łozowicka [7] introduced a one-step QuEChERS extraction and cleanup protocol for 23 sulfonylurea herbicides in cereals using chitin followed by LC-MS, with satisfactory recoveries (70%–120%) reported.

Dispersive solid–liquid extraction (d-SLE), an environmentally friendly cleanup procedure, was introduced by Sun et al. [16]. According to their d-SLE procedure, analytes are adsorbed by sorbents such as C_18_, after which the analytes are eluted from these sorbents using organic solvents. This cleanup procedure was successfully applied to determine the E/Z-fluoxastrobins (broad-spectrum fungicides) in fruits and vegetables, which demonstrated better cleanup and lower matrix effects than the d-SPE. Yao et al. [17] reported that the d-SLE procedure showed lower matrix effects than the d-SPE; however, sensitivity toward some fungicides and insecticides (carbendazim, clothianidin, imidacloprid, prochloraz, thiamethoxam, etc.) following the d-SLE cleanup were not satisfactory due to dilution and the lack of nitrogen blowing and evaporation procedures. However, the cleanup efficacy of d-SLE for the multi-residue analysis of sulfonylurea herbicides has not been studied as this novel technique has only recently appeared.

Herein, we propose that the d-SLE cleanup method can be applied to determine sulfonylurea herbicide residues in strawberries. In this study, we developed and validated a multi-residue analysis technique for the seven sulfonylurea herbicides found in strawberries, namely, azimsulfuron, chlorsulfuron, ethoxysulfuron, flucetosulfuron, halosulfuron-methyl, imazosulfuron, and metazosulfuron, using liquid chromatography-tandem mass spectrometry (LC-MS/MS) in combination with d-SLE. The cleanup efficiency of d-SLE was compared to that of the QuEChERS method.

## 2. Materials and Methods

### 2.1. Chemicals and Reagents

Ammonium acetate (>99.0%), triphenyl phosphate (TPP), formic acid, and magnesium sulfate were purchased from Sigma-Aldrich (St. Louis, MO, USA). A QuEChERS AOAC extraction kit (P/N 5982-7755), QuEChERS d-SPE kits for general fruits and vegetables (P/N 5982-5022), fatty samples (P/N 5982-5122), pigments (P/N 5982-5222), pigments and fats (P/N 5982-5421), and a C_18_ adsorbent (end-capped) were purchased from Agilent Technologies (Santa Clara, CA, USA). All solvents used were of analytical or HPLC grade. The standard herbicide solutions listed in Table 1 were obtained from AccuStandard (New Haven, CT, USA). The purities of all standards exceeded 95%. Herbicide standard stock solutions (each 100 µg mL^−1^) were prepared in acetonitrile. The combined working standard solutions were prepared by serial dilutions of the stock solutions with the same solvent. The stock and working solutions were stored at −20 °C until analyzed.

### 2.2. Sample Preparation

#### 2.2.1. Extraction and d-SLE

For d-SLE, strawberry samples collected from local markets were stored at 4 °C and analyzed within 3 days. Frozen strawberries were homogenized using a commercial grinder and each sample (1 g) was placed in a 15 mL centrifuge tube to which 2 mL of acetonitrile containing 1% formic acid was added as the extraction solvent. The centrifuge tube was vigorously shaken on vortex for 2 min. Then, 150 mg of magnesium sulfate (MgSO_4_) was added and the tube was shaken for 1 min, after which it was centrifuged at 5000 *g* for 10 min at 4 °C. Aliquots (0.2 mL) of the supernatant extract was transferred to 2 mL centrifuge tubes containing different amounts of C_18_ (50, 100, 150, 200, 250, and 300 mg) to which different volumes of water (0.5, 1, 1.5, and 2 mL) were added to the selected amount of C_18_. The tubes were shaken for 1 min and then centrifuged at 15,294 *g* for 3 min. Each supernatant was removed with a 10 mL disposable syringe and 50 mg anhydrous MgSO_4_ was added to remove residual water. Acetonitrile (1 mL) was then added and the tube shaken for 1 min and centrifuged at 15,294 *g* for 3 min. Each supernatant was filtered through a 0.22 μm nylon membrane filter and analyzed by LC-MS/MS.

#### 2.2.2. QuEChERS Extraction and d-SPE

The QuEChERS extraction and d-SPE (QuEChERS-d-SPE) were carried out using the QuEChERS AOAC extraction kit containing 6 g of MgSO_4_ and 1.5 g of sodium acetate. Each sample (15 g) was placed in a 50 mL centrifuge tube to which a 15 mL solution of 1% acetic acid in acetonitrile was added as the extraction solvent. Triphenyl phosphate was spiked directly into the centrifuge tube (as the internal standard) to a concentration of 1 µg mL^−1^. The centrifuge tube was shaken for 1 min. The QuEChERS AOAC extraction kit was then added and the tube was shaken strongly for 10 min, after which the sample was centrifuged at 4000 × *g* for 10 min at 4 °C. Cleanup was performed by d-SPE following analyte extraction. A 1 mL aliquot of the upper layer was transferred to a 2 mL d-SPE tube containing various sorbent mixtures. Five different cleanup procedures were tested, namely, cleanup 1:50 mg primary secondary amine (PSA), and 150 mg MgSO_4_; cleanup 2: 50 mg PSA, 50 mg C_18_, and 150 mg MgSO_4_; cleanup 3: 50 mg PSA, 50 mg GCB, and 150 mg MgSO_4_; cleanup 4: 50 mg PSA, 50 mg C_18_, 50 mg GCB, and 150 mg MgSO_4_; cleanup 5: 50 mg C_18_ and 150 mg MgSO_4_. The tube was tightly closed and vortexed for 1 min, after which it was centrifuged at 15,294 × *g* for 5 min at 4 °C. The extract was filtered through a syringe with a 0.22 μm nylon membrane filter and transferred into an autosampler vial for LC-MS/MS analysis.

### 2.3. LC-MS/MS

Analyses were performed on an Agilent LC 1200 HPLC system (Agilent Technologies, Santa Clara, CA, USA) coupled to a 4000 QTRAP mass spectrometer equipped with a turbo ion-spray ionization source (AB SCIEX, Foster City, CA, USA). Chromatographic separations were achieved using a reversed-phase Cadenza CD-C_18_ HT column (50 × 2.0 mm, 3 μm; Imtakt Company, Kyoto, Japan). The mobile phase consisted of: (A) 5 mM ammonium acetate and 0.1% (*v*/*v*) formic acid in water, and (B) 5 mM ammonium acetate and 0.1% (*v*/*v*) formic acid in methanol. A linear binary mobile phase solvent gradient was used as follows: 95% A at 0 min, 60% A at 0.5 min, 40% A at 1.5 min, 30% A at 2 min, 20% A at 5 min, 0% A at 6–7.5 min, and 95% A at 8–12 min. The flow rate, column temperature and injection volume were 0.3 mL min^−1^, 40 °C, and 2 μL, respectively. The mass spectrometer was operated in the positive-ion ESI mode. The MS/MS was performed using scheduled multiple reaction monitoring (MRM) with the following general settings: curtain gas, 30 psi; ion-source gas (1), 50 psi; ion-source gas (2), 55 psi; source temperature, 400 °C; and ion-spray voltage, 5500 V. The MRM transitions, retention times, collision energies, and declustering potentials of the analytes are summarized in Table 1. The contents of individual herbicides were calculated using the matrix-matched calibration curve.

### 2.4. Validation Study and Matrix Effects

The method was validated following the European Commission SANTE/11813/2017 [18] and ICH/2005/Q2/R1 [19] protocols. To assess linearity, blank extracts were spiked with a multistandard solution at concentrations of 0.005, 0.01, 0.02, 0.05, and 0.1 mg/kg. A coefficient of determination *R*^2^ > 0.99 was acceptable.

The recovery (%) and precision, in terms of repeatability and reproducibility, were determined by repeated analysis of fortified blank samples at three concentrations (0.01, 0.05, and 0.1 mg/kg). The recovery (%) is expressed as: Recovery (%) = (measured concentration)/(spiked concentration) × 100. Repeatability and reproducibility were determined by at least six-replicate analyses on the same day and on different days. Precision is expressed as the relative standard deviation (RSD) of replicate measurements.

The limit of detections (LODs) and limit of quantifications (LOQs) were determined from five independently spiked concentrations of herbicides (0.005, 0.01, 0.02, 0.05, and 0.1 mg/kg). The LODs and LOQs were calculated based on the standard deviations of response and slope, and they are expressed as: LOD = 3.3 σ/s, LOQ = 10 σ/s, where, σ is the standard deviation of the response, and s is the slope of the matrix-matched calibration curve.

To compensate for matrix effects (MEs), we compared the slopes of the pesticide standards in the solvent and in the extracts. The ME (%) is expressed as:

ME (%) = (slope of the calibration curve of the extract/slope of the calibration curve of solvent − 1) × 100.(1)

## 3. Results and Discussion

### 3.1. Optimizing the LC-MS/MS Parameters

The ionizations and fragmentations of the seven sulfonylurea herbicides were studied prior to method validation. The seven sulfonylurea herbicides in strawberries were identified on the basis of the retention times and ion abundances of qualitative and quantitative ions. The positive ESI technique was used for LC-MS/MS. The most intense product ion (*m*/*z*) was used to quantify each herbicide. The selected sulfonylurea herbicides were successfully determined, both qualitatively and quantitatively, using multiple reaction monitoring (MRM). The optimized LC-MS/MS parameters, namely retention times, quantified and qualified ion transitions, declustering potentials, and collision energies for data acquisition, were used to obtain the best MRM transitions (Table 1).

Since the seven sulfonylurea herbicides, which contain sulfonic functional groups, are weak acids according to their pKa values (approximately 3–5) [20], acidic mobile phases provided better retentions and chromatographic separations. The selected herbicides were analyzed using ammonium acetate and formic acid as the mobile phase additives, which improved peak intensities, and promoted the ionization and separation of the analytes [21]. To ensure the highest resolution for all analytes, the gradient conditions were optimized by adjusting the flow rate to 0.3 mL min^-1^ which resulted in an analysis time of 12 min. Well-resolved and separated peaks with good shapes were achieved, as shown in Supplemental Figure S1.

### 3.2. Optimizing the d-SLE Method

We investigated the efficacy of the d-SLE cleanup method, which is capable of enriching analytes and removing the co-eluent through adsorption and desorption procedures. To optimize this method, the recoveries (%) of the seven sulfonylurea herbicides were compared when different amounts of C_18_ and volumes of water were added to the extraction solvent. Acetonitrile containing 1% formic acid has been previously reported to provide the best recoveries for 23 sulfonylurea herbicides [7] and was selected as the extraction solvent in this study. Strawberries contain water-soluble pigments and vitamins, and over 90% moisture. The use of MgSO_4_ in the partition step reduced the volume of the aqueous phase, resulting in the removal of the water-soluble co-eluents.

#### 3.2.1. The Effect of the Amounts of C_18_ on Extraction Efficacy

**Figure 1:**
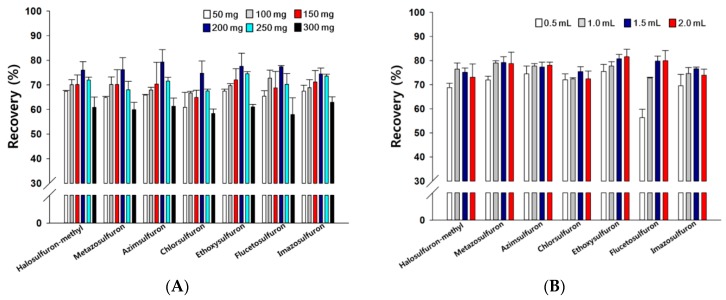
Recoveries as functions of (**A**) the amount of added C_18_ and (**B**) the volume of added water for selected sulfonylurea herbicides in strawberries fortified at 0.05 mg/kg using the dispersive solid–liquid extraction (d-SLE) method. In (**A**), 1.0 mL of water was added to facilitate the adsorption of the analytes in acetonitrile onto the C_18_. In (**B**), 200 mg of C_18_ was used to adsorb the target herbicides.

In order to achieve satisfactory cleanup, we optimized the amount of adsorbent used. The effect of C_18_ in the 50–300 mg range was examined at a fortified level of 0.05 mg/kg. Water (1 mL) was added to promote the adsorption of the analytes in the acetonitrile onto the C_18_. As shown in Figure 1A, the recoveries depend on the sulfonylurea herbicide. It is evident that less than 200 mg of C_18_ provides lower recoveries for all tested herbicides (Figure 1A). Lower recoveries were observed when the amount of C_18_ was increased from 200 to 300 mg, with the lowest recoveries observed with 300 mg of C_18_ (Figure 1A). The optimized amount of C_18_ used in the cleanup step was sufficient to remove matrix interferences, and thus led to an improvement of target analytes detection and their recoveries [22]. Consequently, 200 mg of C_18_ was selected for the adsorption of the target herbicides in this study. Leandro et al. [23] reported that the addition of 200 mg of the C_18_ sorbent led to an effective reduction of nonpolar analytes.

#### 3.2.2. The Volume of Added Water

The volume of the added water, which reduces the proportion of acetonitrile, is a critical factor that significantly affects the retention or adsorption performance of adsorbents toward analytes in a reversed-phase system. Various volumes of water (0.5–2.0 mL) were added to the extract with 200 mg of C_18_. As shown in Figure 1B, the volumes of added water examined do not appear to significantly affect the recoveries (%) of azimsulfuron, chlorsulfuron, and ethoxysulfuron. Increasing the volume of water to 1.5 mL led to increases in the recoveries of some herbicide (Figure 1B); however, the addition of more than 1.5 mL of water resulted in no further changes in the observed recoveries, which is in agreement with the study by Sun et al. [16]. Therefore, adding 1.5 mL of water was used to promote the adsorption of herbicides in further experiments. After removal of the water, 1 mL of acetonitrile was used as the desorption solvent and MgSO_4_ was added to remove trace amounts of residual water.

### 3.3. Method Validation and Matrix Effects

Validation experiments that assess linearities, accuracies, precisions, LODs, and LOQs, were used to evaluate the extraction and cleanup procedure under the optimized d-SLE conditions. Calibration curves were constructed by the matrix-matched standard calibration method at concentrations of 0.005, 0.01, 0.02, 0.05, and 0.1 mg/kg in blank strawberry extracts. As shown in Table 2, satisfactory correlation coefficients (*R*^2^) of 0.9985–0.9994 were obtained for the seven sulfonylurea herbicides, while LODs between 0.001 mg/kg and 0.002 mg/kg were observed. In addition, LOQs between 0.004 mg/kg and 0.005 mg/kg were obtained for the target analytes, with the majority lower than the lowest points of the respective linear ranges. Moreover, the d-SLE method provided a lower LOQ than the MRLs established by the EU and Korea for each sulfonylurea herbicide studied. On the basis of the EU requirements, each LOQ should be lower or equal to the MRL. Recoveries were determined at three fortification levels (0.01, 0.05, and 0.1 mg/kg), with recoveries of 70%–84%, repeatability RSDs of less than 14%, and reproducibility RSDs of less than 14% observed for all herbicides, which are within the quality control criteria prescribed by the SANTE guidelines.

MEs (%) were determined in order to evaluate ion suppression and/or enhancement, which play crucial roles in analyte quantification [24]. An ME of between −20% and 20% is regarded to indicate no matrix effect, while a value outside of this range indicates enhancement (>20%) or suppression (<−20%) [25]. Regarding the strawberry matrix, all tested sulfonylurea herbicides that were cleaned up using d-SLE exhibited no matrix effects (Table 2). Minimizing the ME obtained by removing co-eluted matrix interference can improve chromatographic selectivity. The d-SLE method exhibited a better ME than the d-SPE method, which is due to the high dilution factor [17]. An appropriate dilution factor can reduce the ME to less than 20% [26]. In this study, the d-SLE method provided good MEs with acceptable recoveries at significantly higher dilutions (more than ten times) than those used for the d-SPE method (Table 2).

### 3.4. Comparing d-SLE and QuEChERS-d-SPE

The d-SLE and QuEChERS-d-SPE methods were compared in order to evaluate analyte-extraction and cleanup procedure performance. Acetate-buffered extraction was used in the QuEChERS-d-SPE method because this method has been shown to provide higher and more consistent recoveries for pH-dependent pesticides in fruit and vegetable matrices [27]. PSA, GCB, and C_18_ are the sorbents most commonly used to remove co-extracts from fruits and vegetables during cleanup [28,29,30]; however, the optimal cleanup sorbent depends on the characteristics of the pesticide components and may vary as a consequence. PSA and GCB can adsorb some weakly acidic herbicides, including sulfonylureas [31]. As summarized in Table 3, the recovery rates of most sulfonylurea herbicides using d-SPE with PSA (cleanup 1–4) were found to be less than 70%. The recovery rates were lower using cleanup 3, which uses both GCB and PSA, than cleanup 1 which uses only PSA; similar results were also observed for cleanups 2 (PSA + C_18_) and 4 (PSA + GCB + C_18_). GCB is known to adsorb and retain pesticides with planar structures [27,32,33]. Moreover, the addition of C_18_ (cleanup 4) tended to further reduce the recovery rates of some of the herbicides as compared with d-SPE without C_18_ (Cleanup 3). Lower extraction efficacies were observed with C_18_ when the aqueous phase was not completely removed by phase separation [22]. The reduced recoveries (%) observed with the d-SPE methods (Table 3) is partially ascribable to the use of GCB and PSA, and/or a combination of GCB, PSA, and C_18_. On the other hand, cleanup 5, which only used C_18_, met the requirements of accuracy (74.1%–87.2%), which is ascribable to the C_18_ sorbent having the lowest affinity for acidic herbicides as compared with PSA and GCB, and excellent performance during extract purification [31]. Kaczyński et al. [7] reported a method for extracting acidic herbicides with C_18_ cleanup; their method exhibited recoveries of between 65% and 89% for sulfonylurea herbicides, which are consistent with our results (Table 3). Lee et al. [8] also reported that C_18_ cleanup afforded the best recoveries for some sulfonylurea herbicides in brown rice and rice straw.

The d-SLE method effectively removed impurities and pigments. The strawberry samples purified by d-SLE were visually colorless as compared with those purified by d-SPE. The recoveries using the d-SLE method were 70.4%–83.7%, which are comparable with the C_18_ cleanup recoveries of between 74.1% and 87.2% at fortification levels of 0.01, 0.05, and 0.5 mg/kg. The results in this study demonstrate that the seven sulfonylurea herbicides meet the recovery and %RSD requirements using d-SLE with C_18_ as an adsorbing material in the absence of an extraction salt. Therefore, this study suggests that d-SLE is a comparable cleanup method for the effective analysis of sulfonylurea herbicides.

QuEChERS extraction was carried out using the AOAC (6 g MgSO_4_ and 1.5 g sodium acetate) extraction kit. Cleanups were performed as follows: Cleanup 1, 50 mg PSA; cleanup 2, 50 mg PSA + 50 mg C_18_; cleanup 3, 50 mg PSA + 50 mg GCB; cleanup 4, 50 mg PSA + 50 mg C_18_ + 50 mg GCB; and cleanup 5, 50 mg C_18_.

### 3.5. Applying the Analytical Method

Table 4 lists the analysis data for sulfonylurea herbicides in ten real strawberry samples collected from local markets in Wanju, Korea. Although the selected herbicides exhibited no MEs after d-SLE cleanup, matrix-matched calibration curves were prepared for more accurate quantification. All tested herbicides were present below the LODs and MRL levels recommended by Korea [5] and the EU [4] using d-SLE cleanup followed by LC-MS/MS. According to these results, the optimized d-SLE method combined with LC-MS/MS is suitable for determining multiple sulfonylurea herbicide residues in strawberry samples. 

## 4. Conclusions

We demonstrated that an optimized d-SLE cleanup procedure in combination with LC-MS/MS can be used to determine multiple sulfonylurea herbicides residues in strawberries. The d-SLE method positively influenced strawberry-extract cleanup, providing good linearity, precision, and accuracy for each sulfonylurea herbicide examined. The LOQs are compliant with Korea and EU MRLs. The d-SLE method in combination with LC-MS/MS was subsequently applied to monitor herbicide residues in ten strawberry samples, with no MRL exceedances observed for any tested sample. We suggest that d-SLE using C_18_ as the adsorbent is an alternative method for quantitatively analyzing sulfonylurea herbicides that remain in strawberries. However, further study is needed in order to determine the applicability of the d-SLE method to other crops, such as rice and wheat, among others, where sulfonylurea herbicides are mainly used.

## Figures and Tables

**Table 1:** LC-MS/MS parameter values used during the detection of selected sulfonylurea herbicides.

Sulfonylurea Herbicides ^a^	R.T. (min)	Precursor Ion (*m*/*z*)	Quantification Transition (*m*/*z*)	D.P. (V)	C.E. (V)	Confirmatory Transition (*m*/*z*)	D.P. (V)	C.E. (V)
Azimsulfuron	4.6	425.0	182.1	61	23	156.1	61	45
Chlorsulfuron	4.3	410.9	149.0	66	27	119.1	66	53
Ethoxysulfuron	5.3	388.0	167.1	71	21	204.9	71	33
Flucetosulfuron	4.9	358.0	141.0	71	23	167.1	71	25
Halosulfuron-methyl	5.6	435.1	182.1	51	35	138.9	51	71
Imazosulfuron	5.2	399.0	217.9	66	33	260.9	66	21
Metazosulfuron	5.0	476.0	181.8	91	33	295.1	91	25

^a^ The mass spectra for all of the pesticides tested was obtained using the positive ion mode. R.T., retention time; D.P., declustering potential; and C.E., collision energies.

**Table 2:** Matrix-effect and method-validation data for selected sulfonylurea herbicides in strawberries using the d-SLE method with LC-MS/MS.

Sulfonylurea Herbicides	Matrix Effect (%)	Linearity (*R*^2^)	LOD (mg/kg)	LOQ (mg/kg)	Recovery (%)			Repeatability, %RSD			Within-laboratory Reproducibility, %RSD		
					0.01	0.05	0.1	0.01	0.05	0.1	0.01	0.05	0.1
					mg/kg			mg/kg			mg/kg		
Azimsulfuron	−16.0	0.9988	0.001	0.004	79.7	76.6	75.8	4.5	2.0	3.7	7.3	4.1	11.1
Chlorsulfuron	1.0	0.9991	0.001	0.005	77.5	76.0	83.2	12.2	0.2	0.8	13.6	2.4	8.6
Ethoxysulfuron	−4.5	0.9994	0.001	0.004	79.8	73.7	82.0	8.6	0.9	4.0	6.1	2.2	8.3
Flucetosulfuron	0.9	0.9985	0.002	0.005	75.7	83.7	70.4	13.8	13.0	0.6	12.5	10.0	13.8
Halosulfuron-methyl	−11.2	0.9993	0.002	0.005	77.9	74.9	70.4	7.0	1.4	2.7	8.0	1.0	9.2
Imazosulfuron	−9.3	0.9988	0.002	0.005	81.8	76.2	75.3	8.6	2.4	0.4	8.2	1.8	10.0
Metazosulfuron	−14.1	0.9989	0.001	0.004	78.2	77.0	79.9	8.4	3.7	2.1	8.6	3.0	10.8

LOD, limit of detection. LOQ, limit of quantification. RSD, relative standard deviation.

**Table 3:** Comparing selected sulfonylurea herbicide recoveries from strawberries using five dispersive solid phase extraction (d-SPE) cleanup procedures and the optimized d-SLE method.

Sulfonylurea Herbicides	Fortification Level (mg/kg)	Mean Recovery (%)					
		Cleanup 1	Cleanup 2	Cleanup 3	Cleanup 4	Cleanup 5	d-SLE ^a^
Azimsulfuron	0.01	72.3	56.9	28.8	29.9	82.1	79.7
	0.05	62.0	62.2	29.1	22.3	78.2	76.6
	0.1	58.1	66.0	44.3	24.3	85.9	75.8
Chlorsulfuron	0.01	38.8	43.6	33.4	33.1	79.9	77.5
	0.05	47.8	48.5	36.9	27.3	74.8	76.0
	0.1	42.7	49.8	46.2	31.4	79.8	83.2
Ethoxysulfuron	0.01	64.1	52.0	3.8	3.2	86.5	79.8
	0.05	50.2	56.7	3.7	2.8	81.7	73.7
	0.1	41.7	54.8	9.0	2.7	86.3	82.0
Flucetosulfuron	0.01	72.0	62.4	24.4	23.2	82.1	75.7
	0.05	64.5	67.9	22.6	23.9	75.0	83.7
	0.1	55.7	70.8	39.7	20.5	82.9	70.4
Halosulfuron-methyl	0.01	54.5	53.4	36.0	33.1	74.1	77.9
	0.05	50.3	55.1	42.5	27.6	75.3	74.9
	0.1	42.0	54.7	57.4	28.8	80.8	70.4
Imazosulfuron	0.01	68.4	57.3	20.5	19.2	87.2	81.8
	0.05	57.3	60.4	18.8	15.0	83.8	76.2
	0.1	52.3	61.2	31.7	15.4	87.1	75.3
Metazosulfuron	0.01	42.7	37.8	24.0	21.1	87.0	78.2
	0.05	37.2	41.1	30.3	17.2	83.7	77.0
	0.1	29.2	42.4	46.9	17.7	84.4	79.9

^a^ C_18_ (200 mg) and water (1.5 mL) were added during the determination of sulfonylurea herbicides in strawberries by d-SLE.

**Table 4:** Applying the d-SLE method to ten strawberry samples.

Sulfonylurea Herbicides	Korea MRLs ^a^ (mg/kg)	EU-MRLs ^b^ (mg/kg)	No. of Samples	
			>LOQ	>LOD
Azimsulfuron	0.01	0.01		
Chlorsulfuron	0.01	0.05		
Ethoxysulfuron	0.01	0.01		
Flucetosulfuron	0.01	NA		
Halosulfuron-methyl	0.01	0.01		
Imazosulfuron	0.01	0.01		
Metazosulfuron	0.01	NA		

^a^ Maximum residue limit (MRL) on Food Code [5]. ^b^ European Union maximum residue level [4]. NA, MRL not currently available for strawberry analyzed. LOD, limit of detection. LOQ, limit of quantification.

## Supplementary Materials

The following are available online at https://www.mdpi.com/2304-8158/8/7/273/s1, Figure S1: Dispersive solid–liquid extraction coupled with LC-MS/MS for the determination of sulfonylurea herbicides in strawberries.
