# Supplementary material for: Dispersive Solid–Liquid Extraction Coupled with LC-MS/MS for the Determination of Sulfonylurea Herbicides in Strawberries
Source: Foods. 2019 Jul 22;8(7):273. doi: 10.3390/foods8070273 (PMC6678714; doi:10.3390/foods8070273)
Supplement: Supplementary file 1 [file foods-08-00273-s001.pdf]

Supplementary Material

Dispersive solid-liquid extraction coupled with LC-MS/MS for the determination of sulfonylurea herbicides in strawberries

Song *et al.*

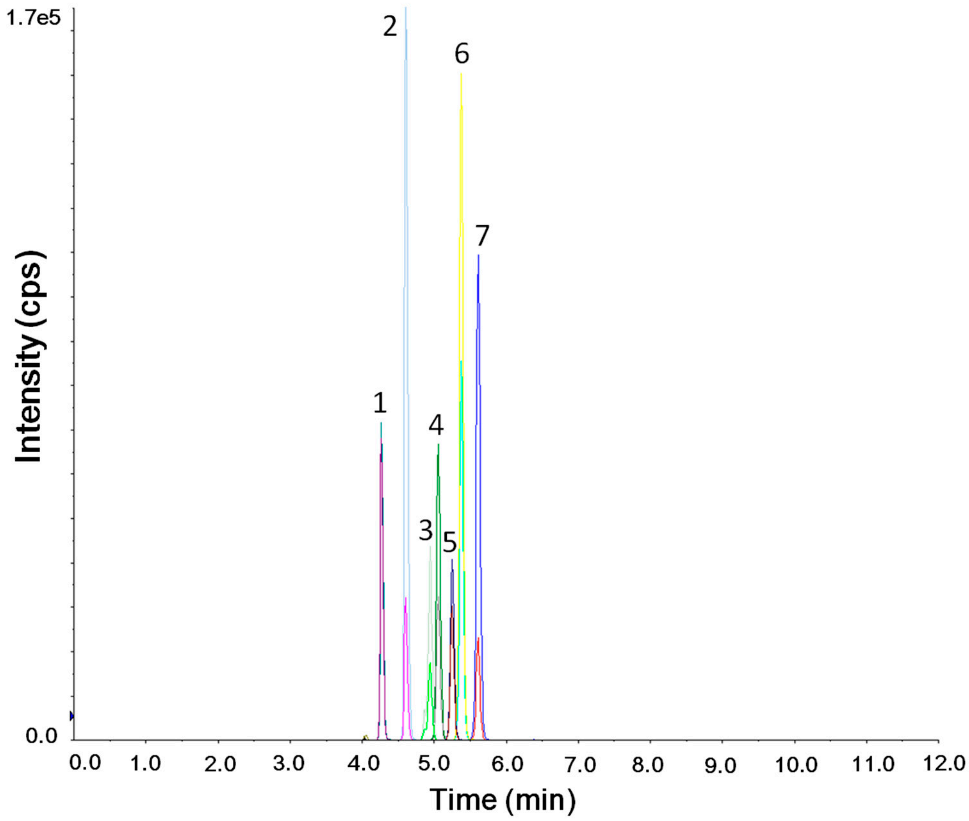

Figure S1. The multiple reaction monitoring chromatogram of 7 sulfonylurea herbicides at the optimized LC–MS/MS conditions. Peaks: 1, chlorsulfuron; 2, azimsulfuron; 3, flucetosulfuron; 4, metazosulfuron; 5, imazosulfuron; 6, ethoxysulfuron; 7, halosulfuron-methyl.
